# Raw lacquer-associated familial chronic myelomonocytic leukemia with multi-hit *TET2* mutations

**DOI:** 10.3389/fonc.2025.1605369

**Published:** 2025-08-22

**Authors:** Yang Zhang, Yujiao Luo, Hongling Peng, Yafei Yin, Guangsen Zhang

**Affiliations:** ^1^ Department of Oncology, The Second Xiangya Hospital, Central South University, Changsha, Hunan, China; ^2^ Department of Hematology, Institute of Molecular Hematology, The Second Xiangya Hospital, Central South University, Changsha, Hunan, China; ^3^ Department of Hematology, The Affiliated Changsha Central Hospital, Hengyang Medical School, University of South China, Changsha, Hunan, China

**Keywords:** raw lacquer, chronic myelomonocytic leukemia, familial, *TET2* mutation, Runx1, N-ras

## Abstract

**Background:**

To explore the potential association between long-term exposure to raw lacquer and the development of chronic myelomonocytic leukemia (CMML).

**Methods:**

We analyzed the clinical and hematological characteristics of an elderly couple with CMML. Whole-exome sequencing (WES) was performed to identify relevant gene variants, with a focus on *TET2* mutation status.

**Results:**

Two unrelated CMML patients within the same family, both with over 40 years of raw lacquer exposure, developed CMML. WES revealed that both patients harbored multi-hit *TET2* gene mutations and lacked *ASXL1* mutations. Both demonstrated relative sensitivity to hydroxyurea or hypomethylating agent (HMA) therapy. Unaffected family members lacked significant raw lacquer exposure.

**Conclusions:**

Long-term exposure to raw lacquer may be associated with the onset of familial CMML. CMML patients with multi-hit *TET2* mutations in the absence of *ASXL1* mutations may have a favorable prognosis.

## Introduction

A familial cluster of chronic myelomonocytic leukemia (CMML) is presented, involving a non-consanguineous couple diagnosed with CMML at approximately 75 years of age, with diagnoses occurring 2 years apart. Both patients exhibited identical clinical and morphological features. A significant environmental factor shared by the couple was chronic exposure to raw Chinese lacquer for over 40 years, suggesting that it may be an extrinsic pathogenic factor for familial CMML. Whole-exome sequencing (WES) identified multipoint mutations in the *TET2* gene in both patients, along with mutations in transcription factors *RUNX1* and *NRAS*. This suggests that *TET2* may be a molecular target of urushiol and brenzcatechin, the main components of raw lacquer. As no other specific extrinsic etiologic factors were identified, chronic raw lacquer exposure may be associated with an increased risk of CMML and its familial aggregation.

## Case reports

### Case 1

**Figure 1:**
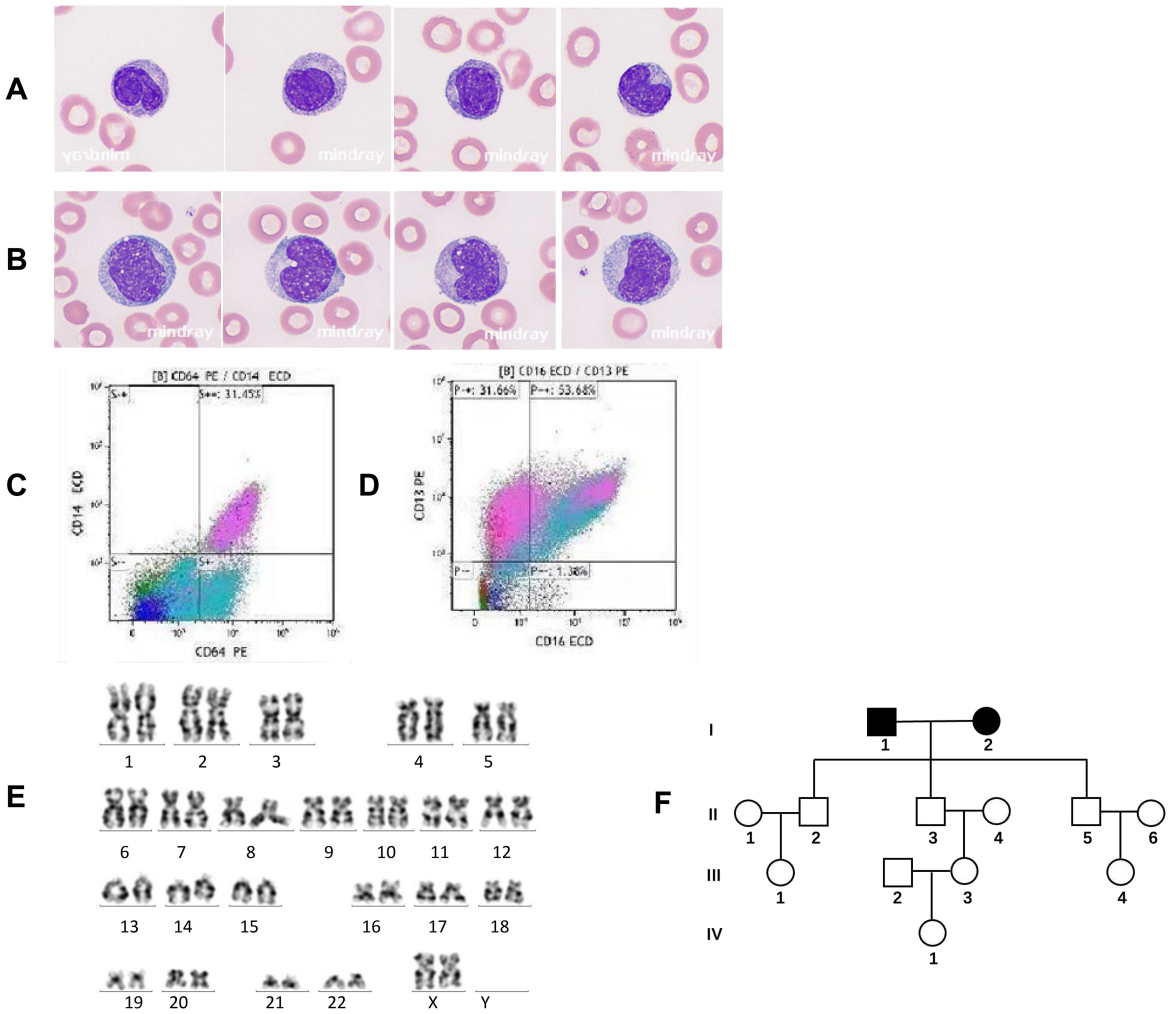
Peripheral blood morphology, cytogenetics, and flow cytometry immunophenotyping in chronic myelomonocytic leukemia (CMML). **(A)** Peripheral blood smear of Case 1 with familial CMML indicating mature or immature monocytes with smaller nuclei and a nuclear/plasma ratio Wright–Giemsa, ×1,000. **(B)** Peripheral blood smear of a control case with sporadic CMML showing immature or mature monocytes with larger nuclei and the nuclear/plasma ratio. Wright–Giemsa, ×1,000. **(C, D)** Immunophenotyping of monocytes by flow cytometry demonstrating monocyte fractions [CD14+CD16+ (non-classical)] in Case 1 with familial CMML. **(E)** The karyotype of Case 1 (46,XX) with familial CMML. **(F)** Pedigree of the familial CMML. Apart from the married couple (I1 and I2) diagnosed with CMML, all other members in the pedigree were unaffected.

A 75-year-old woman was referred to the Department of Hematology, The Second XiangYa Hospital, Changsha, Hunan, for evaluation of leukocytosis [white blood cell (WBC) count 26.15 × 10^9^/L] persisting for 3 weeks. The initial presentation involved abdominal pain and weakness. Peripheral blood analysis revealed monocytosis (24.2%, absolute monocyte count 6.33 × 10^9^/L), anemia (hemoglobin 79 g/L), and thrombocytopenia (platelets 10 × 10^9^/L). Bone marrow (BM) aspiration showed hypercellularity with 25% monocytes (23% mature monocytes and 2% monoblasts). Flow cytometry immunophenotyping was positive for CD13, CD33, CD14, CD16, CD64, HLA-DR, CD4, CD11b, and CD36, but negative for CD34, CD117, CD15, and CD56. Cytogenetic analysis revealed a normal karyotype (**Figures 1C–E**). She was diagnosed with myeloid proliferative CMML (MP-CMML) and classified as high-risk [Mayo Molecular Model (MMM) = 3 risk factors] (1). Treatment with subcutaneous azacitidine (100 mg/day for 7 days per month) was initiated. Hematological complete remission was achieved after four cycles.

The patient’s medical history was significant only for occasional contact dermatitis. Her husband worked as a raw lacquer producer and painter. They were exposed to raw lacquer for more than 40 years. Physical examination at presentation revealed moderate anemia without palpable superficial lymphadenopathy or hepatosplenomegaly. Laboratory findings included hemoglobin 77 g/L, WBC 7.79 × 10^9^/L with monocytes 31.6% (absolute count 2.46 × 10^9^/L), and platelets 22 × 10^9^/L. Virological screening (Epstein-Barr Virus DNA (EBV-DNA), Cytomegalovirus (CMV), coxsackievirus, rubella, Herpes simplex virustype 1 (HSV-1), *Toxoplasma*, and HIV) was negative. Serum levels of trace elements and heavy metals (Zn, Pb, As, Ti, Cd, Cu, Se, Fe, Ni, Co, Pt, and Mn) were within normal ranges. Exclusionary testing was negative for *BCR::ABL1*, *PDGFRα*, and *PDGFRβ* fusion genes, as well as *JAK2 V617F*, *MPL*, and *CALR* mutations.

### Case 2 (husband of case 1)

A 77-year-old man presented on May 17, 2021, with pallor, weakness, and exertional dyspnea. Peripheral blood analysis showed leukocytosis (WBC 34.43 × 10^9^/L) with monocytosis (35.9%, absolute monocyte count 12.36 × 10^9^/L), anemia (hemoglobin 96 g/L), and a platelet count of 136 × 10^9^/L. Bone marrow morphology was consistent with CMML. WES covering 242 hematological malignancy-related genes was performed on peripheral blood mononuclear cells (PBMCs) (Chigene Medical Laboratory, Beijing, China). His past medical history was unremarkable except for his occupation as a raw lacquer producer and painter for over 40 years in a private workshop, during which he experienced several episodes of lacquer contact dermatitis. He cohabited with his wife (Case 1) for over 55 years, sharing the lacquer exposure for >40 years. His diet and other living conditions were comparable to those of nearby residents. His two sons and one daughter had lived with them for less than 15 years and showed no hematological abnormalities on examination. The family pedigree is shown in **Figure 1F**. He was diagnosed with MP-CMML and managed with low-dose hydroxyurea for leukocyte control. His most recent blood tests showed hemoglobin 73 g/L, WBC 22.83 × 10^9^/L, monocytes 38.2% (absolute count 8.71 × 10^9^/L), and platelets 80 × 10^9^/L. He remains alive despite having the disease.

### Control case (sporadic CMML)

For comparison, a 68-year-old retired man with no history of raw lacquer or related chemical exposure was evaluated on August 26, 2023, for a 3-month history of pallor and weakness. Blood tests revealed leukocytosis (WBC 47.69 × 10^9^/L) with monocytosis (30.7%, absolute monocyte count 14.64 × 10^9^/L), anemia (hemoglobin 89 g/L), and thrombocytopenia (platelets 52 × 10^9^/L). Bone marrow morphology confirmed CMML. He was diagnosed with MP-CMML. Clinical features and hematological parameters of this sporadic case and the familial cases are summarized in **Table 1**. He received standard hypomethylating agent (HMA) therapy (azacitidine: 100 mg/day, days 1–7/month), achieving incomplete hematological remission after five cycles. Due to developing thrombocytopenia with HMA, low-dose hydroxyurea was used for leukocyte control.

**Table 1:** The clinical complaints of CMML patients and CBC data.

Cases	Age (year)	Sex	Diagnosis	Complaints	WBC (×10^9^)	Monocyte %	Monocyte count (×10^9^)	Hemoglobin (g/L)	Platelet count (×10^9^)
Case 1 (familial)	75	Female	*MP-CMML	Weakness, abdominal pain	26.15	24.2	6.33	79	10
Case 2 (familial)	77	Male	MP-CMML	Pallor, weakness, dyspnea	34.43	35.9	12.36	96	136
Case 3 (sporadic)	68	Male	MP-CMML	Pallor, weakness	47.69	30.7	14.64	89	52

WBC, white blood cell; CMML, chronic myelomonocytic leukemia; *MP-CMML, myeloid proliferative CMML; CBC, Complete Blood Count.

## Methods

Two familial CMML patients and one sporadic CMML patient were included after providing informed written consent in accordance with the Declaration of Helsinki. PBMNCs were obtained at diagnosis. The study was approved by the Institutional Ethics Committee of the Second Xiang-Ya Hospital, Central South University.

Genomic DNA was extracted from fresh PBMNCs. WES was performed by Kindstar Global Company (Wuhan, China). Sequencing achieved 99.59% coverage at 20x depth, with an average sequencing depth ≥1,000x. Captured libraries were sequenced on an Illumina NextSeq 550 platform.

## Results

**Figure 2:**
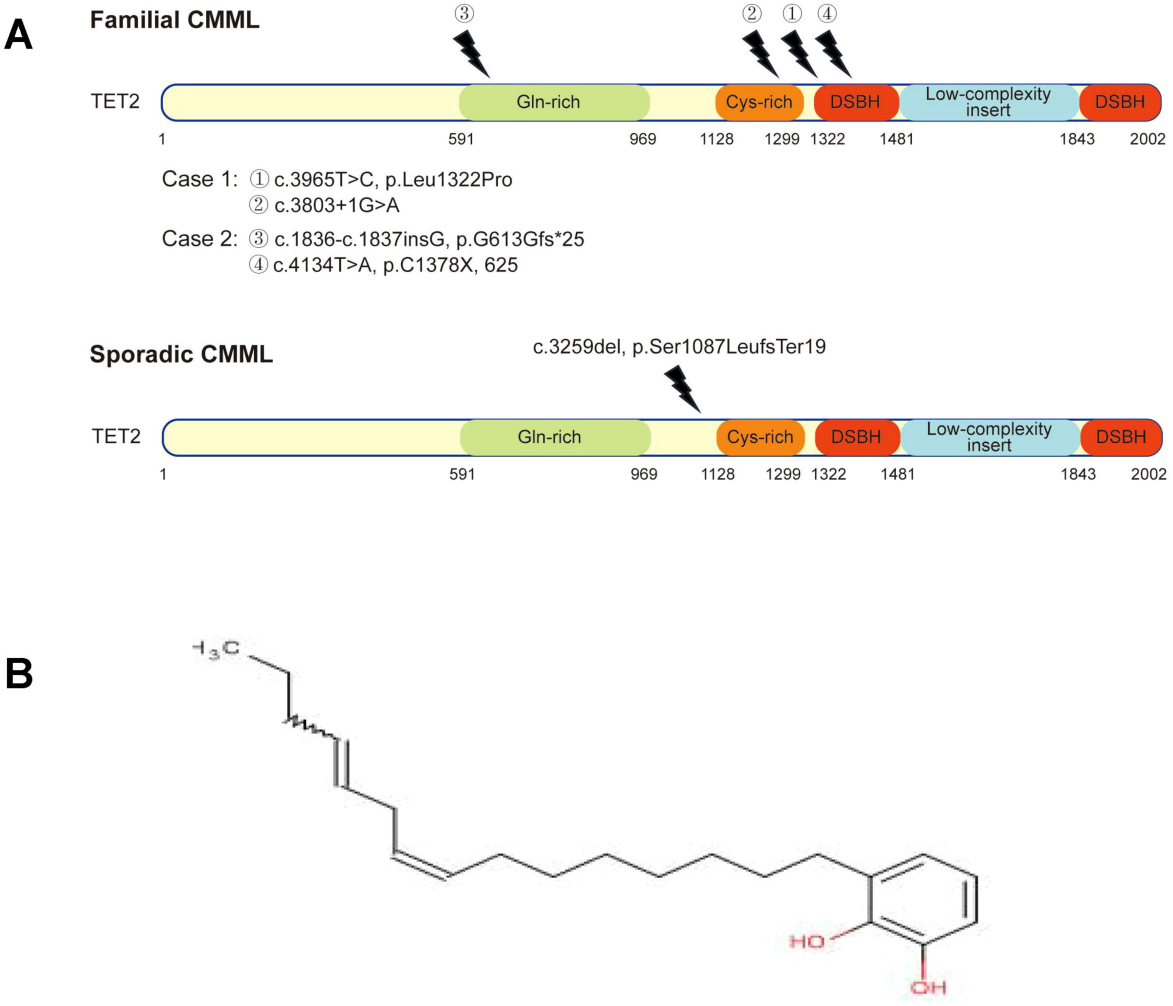
Schematic representation of *TET2* mutations. **(A)** Top panel: mutant sites on TET2 in Case 1 and Case 2 with familial chronic myelomonocytic leukemia (CMML). DSBH, double-stranded β-helix domain. **(A)** Bottom panel: control case with sporadic CMML shows a novel mutation in the *TET2* gene, which is not different from that of TET2 mutation in familial CMML. **(B)** The chemical structure of urushiol.

WES of Case 1 (familial CMML) revealed three somatic mutations: *RUNX1* c.928del [p.Met310Ter; variant allele frequency (VAF), 42.3%], *SRSF2* c.284C>A (p.Pro95His; VAF, 45%), and multi-hit *TET2* mutations [*TET2* c.3803 + 1G>A, a splice site mutation (VAF, 46.6%) and c.3965T>C (p.Leu1322Pro), a missense mutation (VAF, 44.9%)]. The mutant *TET2* loci were located in the Cys-rich domain and double-stranded β-helix (DSBH) domain (**Figure 2A**, top panel), consistent with previous reports (2).

WES of Case 2 (covering 242 genes) identified an *NRAS* mutation (c.35G>T, p.Gly12Val; VAF, 44.0%) and multi-hit *TET2* mutations [c.1836_1837insG, p.Gly613fs*25 (frameshift); VAF, 48%; and c.4134T>A, p.Cys1378*; VAF, 44%]. Both *TET2* mutation sites represent novel loci in the context of CMML (**Figure 2A**, top panel).

WES of the sporadic CMML control case identified five mutations: *ASXL1*, *NRAS*, *RUNX1*, *SRSF2*, and *TET2* (c.3259del, p.Ser1087LeufsTer19; VAF, 84.1%). The *TET2* mutation site is also novel (**Figure 2A**, bottom panel) (2). Genetic mutations and VAFs for all three patients are summarized in **Table 2**.

**Table 2:** Gene mutation types and VAF in familial CMML or sporadic CMML.

Cases	Diagnosis	Gene types	VAF	TET2 mutational state
Case 1 (familial)	MP-CMML	RUNX1 c.928del; p.	42.3%	
		SRSF2 c.284C>A; p.	45.0%	
		TET2 c.3083 + 1G>A; a splice site mutation	46.6%	Multi-hit TET2; c ≥ 2 TET2 mutations
		TET2 c.3965T>C; p.Leu1322Pro	44.9%	
Case 2 (familial)	MP-CMML	NRAS c.35G>T; p.Gly12Val	44.0%	
		TET2 c.1836_c1837 ins G; p.G613Gs*25	48.0%	Multi-hit TET2
		TET2 c.4134T>A; p.c1378X625	44.0%	
Case 3 (sporadic)	MP-CMML	NRAS c.35G>T; p.Gly12Val	43.7%	
		ASYL1 c.1934dup; p.Gly646Trp Ter12	40.3%	
		RUNX1 c.837G>A; p.Trp279Ter	1.2%	
		TET2 c.325p del; p.Ser1087Leufs Ter19	84.1%	Single-hit TET2
		SRSF2 c.284C>A; p.Pro95His	44.6%	

VAF, variant allele frequency; CMML, chronic myelomonocytic leukemia; MP-CMML, myeloid proliferative CMML.

Recent analyses of large CMML cohorts indicate that *TET2* mutations without concurrent *ASXL1* mutations (*ASXL1*WT/*TET2*mut) confer a favorable impact on overall survival (OS) (2, 3). Both familial CMML cases exhibited *ASXL1*WT/*TET2*mut status, while the control case was *ASXL1*mut*TET2*mut, suggesting a potentially poorer prognosis for the latter. This difference is also reflected in the initial response to HMA; the control case was intolerant to HMA and developed therapy-related thrombocytopenia. Morphologically, monocytes from the control case displayed a larger nucleus and a higher nuclear–cytoplasmic ratio (**Figure 1B**) compared to those of Case 1 (**Figure 1A**), suggesting higher proliferative activity.

## Discussion

While certain chemotherapeutic agents are well-established causes of therapy-related leukemia, exposure to environmental agents like benzene and petroleum products has been linked to an increased risk of Acute Myelogenous Leukemia (AML) (4, 5). However, the etiology of CMML remains poorly defined. A case–control study specifically examining CMML did not find an association with benzene exposure (6). Associations between CMML and other chemical substances have not been previously reported.

In this familial cluster of two non-consanguineous individuals developing CMML within 2 years, long-term occupational exposure to raw lacquer emerged as a plausible major risk factor after excluding other potential causes (specific dietary habits, heavy metal poisoning, and viral infection). This hypothesis is supported by 1) a shared 40-year history of occupational raw lacquer exposure; 2) recurrent raw lacquer contact dermatitis in both individuals; 3) the absence of hematological abnormalities in their children, who lacked significant exposure; and 4) lack of evidence for genetic predisposition, viral triggers, or heavy metal poisoning.

Raw lacquer’s primary component is urushiol, a mixture of brenzcatechin derivatives with unsaturated side chains (**Figure 2B**). Urushiol is a well-known cause of contact dermatitis (7, 8), but its association with CMML, particularly in a familial aggregation pattern, has not been reported. Contact dermatitis involves the activation of monocytes or Langerhans-like cells and inflammation (9, 10). It is conceivable that chronic, long-term stimulation of the monocyte system by such agents could promote clonal monocyte proliferation and potentially leukemic evolution.

Reports of familial AML are rare and often syndromic (e.g., Down syndrome). Familial platelet disorder with predisposition to AML (FPD/AML), caused by germline *RUNX1* mutations (*RUNX1*-FPD), has been described in approximately 11 families (11). Case 1 harbored a somatic *RUNX1* mutation (c.928del, p.Met310Ter) distinct from the 62 mutations reported in FPD/AML (12). As her husband lacked germline or somatic RUNX1 mutations and had no history of thrombocytopenia, a diagnosis of RUNX1-FPD or RUNX1-related CMML was not supported. In Case 2, we identified a mutation in the N-RAS gene. It has been reported that approximately 35% of patients with CMML have point mutations in the K-RAS or N-RAS gene, which can result in leukemic transformation events (13). Patel BJ et al. demonstrated that the disease progression of CMML is associated with TET2 and RAS mutations (14). Whether the somatic *RUNX1* mutation in Case 1 or the *NRAS* mutation in Case 2 resulted from DNA damage induced by chronic urushiol exposure warrants further investigation.

*TET2* is the most frequently mutated gene in CMML (~61%) (1, 3, 15), and multiple *TET2* mutations per patient are common at diagnosis (2, 15, 16). The clinical significance lies in the association of multiple *TET2* mutations with older age and improved survival in the absence of *ASXL1* mutations (3, 17). Multihit *TET2* mutations (≥2 mutations) have been proposed as a molecular marker for differentiating oligomonocytic CMML (OM-CMML) from classical CMML (17). The current model suggests that initiating driver mutations often involve *TET2* in hematopoietic stem cells (HSCs) (18), followed by secondary mutations (including additional *TET2* or *SRSF2* mutations) at the myeloid progenitor level, accelerating differentiation toward granulocyte–monocyte progenitors (GMPs) and clonal monocytosis (18). Furthermore, early clonal dominance of TET2 has been described as a unique finding in CMML (19). Loss-of-function *TET2* mutations lead to DNA hypermethylation, altered gene expression, and aberrant monocytic differentiation (19, 20).

In our cases, both patients exhibited multi-hit *TET2* mutations with identical mutation numbers but distinct sites. The VAF of these *TET2* mutations (44%–48%, mean 45.88%) was significantly lower than that observed in the sporadic control case (84.1%). In the control case, although there was a single-hit TET2 mutation, the VAF was significantly increased. This increase of more than 55% could also be classified as multi-hit TET2 mutations (20), which meant that a biallelic alteration due to loss of heterozygosity also reflected the increase of clonal tumor load. Because of the concomitant *ASXL1* mutation and high mutant *TET2* VAF in the control case, he showed insensitivity to demethylating treatment. The clinical implications of these findings may include 1) advanced age at onset for familial CMML (75 and 77 years old); 2) the absence of *ASXL1* mutation, suggesting a relatively favorable prognosis; and 3) multi-hit *TET2* mutations combined with relatively low VAF values, potentially correlating with good sensitivity to HMA or hydroxyurea therapy observed in both patients. Whether chronic urushiol exposure directly drives *TET2* mutations requires further experimental validation and additional case studies.

## Conclusion

We report the first familial cluster of CMML and preliminarily identify long-term raw lacquer exposure as a potential pathogenic factor. Both affected individuals harbored multi-hit *TET2* mutations and demonstrated a favorable response to HMA or hydroxyurea therapy.

## Data Availability

All date generated or analyzed during this study are included in this published article.
